# Novel transposable elements from *Anopheles gambiae*

**DOI:** 10.1186/1471-2164-12-260

**Published:** 2011-05-23

**Authors:** Rita D Fernández-Medina, Cláudio J Struchiner, José MC Ribeiro

**Affiliations:** 1 Fundação Oswaldo Cruz, Escola Nacional de Saúde Pública Sergio Arouca, Av. Brasil, 4365. 21040 360. Rio de Janeiro, Brazil; 2 Instituto de Medicina Social, Universidade do Estado de Rio de Janeiro, Rio de Janeiro, Brazil; 3 Section of Vector Biology, Laboratory of Malaria and Vector Research, National Institute of Allergy and Infectious Diseases, National Institutes of Health, Rockville, MD 20852, USA

## Abstract

**Background:**

Transposable elements (TEs) are DNA sequences, present in the genome of most eukaryotic organisms that hold the key characteristic of being able to mobilize and increase their copy number within chromosomes. These elements are important for eukaryotic genome structure and evolution and lately have been considered as potential drivers for introducing transgenes into pathogen-transmitting insects as a means to control vector-borne diseases. The aim of this work was to catalog the diversity and abundance of TEs within the *Anopheles gambiae *genome using the PILER tool and to consolidate a database in the form of a hyperlinked spreadsheet containing detailed and readily available information about the TEs present in the genome of *An. gambiae*.

**Results:**

Here we present the spreadsheet named AnoTExcel that constitutes a database with detailed information on most of the repetitive elements present in the genome of the mosquito. Despite previous work on this topic, our approach permitted the identification and characterization both of previously described and novel TEs that are further described in detailed.

**Conclusions:**

Identification and characterization of TEs in a given genome is important as a way to understand the diversity and evolution of the whole set of TEs present in a given species. This work contributes to a better understanding of the landscape of TEs present in the mosquito genome. It also presents a novel platform for the identification, analysis, and characterization of TEs on sequenced genomes.

## Background

Transposable elements (TEs) are DNA sequences that can move within a cell's genome from one chromosomal location to another by a reaction called transposition, augmenting in this way their own representation within genomes. Once having infected the germline of an organism, they can also spread through populations. These elements are ubiquitous in eukaryotes, accounting in certain species for a high proportion of their genomic DNA; for instance, approximately 50% of the human genome [1] and up to 80% of the maize genome [2] are composed of TEs. In *Anopheles gambiae*, the transmitting vector of the malaria parasite, these elements constitute 16% of the euchromatin and up to 60% of the heterochromatin of the genome [3]. These elements have quite simple genetic structures, basically containing genes involved in their transposition--which is generally independent from the cellular replicative machinery--and their flanking recognition sites. They can be seen as genomic infectious agents and due to this characteristic were originally considered as selfish DNA [4]; however, as more information was gathered about TE biology, their relevance as important players in evolution and maintenance of eukaryotic genomes has been emphasized [5,6].

TEs can replicate via an RNA or a DNA intermediate and have been traditionally classified accordingly into two classes (I and II) [7-9]. Class I elements, also known as retrotransposons, depend on a reverse transcription step. They are nearly identical to retroviruses in structure and are related phylogenetically [10]. They contain a gag-like gene, a pol-gene (which generally contains domains for reverse transcriptase, RnaseH, integrase, protease, and endonuclease) and in certain cases a third open reading frame (ORF) coding for an env-like protein [8]. Class I is subsequently classified into five orders based on major differences in their insertion mechanism as well as overall genetic organization [11]. Class II elements are genetically and structurally more homogenous and simple. Their basic genetic structure consists of a transposase gene flanked by terminal inverted repeats (TIRs), which are fundamental for their recognition by transposases and further mobilization. This class has been classified into two subclasses, which are subsequently grouped into different orders [11]. The classification of Class II TEs based in their genetic differences permitted a further classification in superfamilies and families.

The proportion of the different classes, orders, and superfamilies of TEs in a given genome varies in different species; some harbor very abundant but few families of TEs as mammals, where Class I non-long terminal repeats (NLTRs) predominate [12,13], while others contain many different yet less abundant families. These differences have been related to the C-value paradox [14], which refers to the huge variations in genome size of eukaryotic organisms despite similar levels of organism complexity [15]. For instance, the large differences observed in genome size between *An. gambiae *(286 Mb) and *Aedes aegypti *(1.3 Gb) is determined by their differential content of TEs.

On the other hand, TEs can be classified as autonomous and non-autonomous based on their ability to perform their own transposition. Moreover, within these two categories, TEs can be also classified as active or inactive based in their actual mobility, which depends on several factors, among which the presence of an active, autonomous element of the same family--or close enough to be able to mobilize it--is fundamental.

An important aspect of the study of TEs in a given genome is the understanding of their abundance and diversity as well as the classification and organization of the elements. Currently, Repbase is the reference collection of repetitive elements from most eukaryotic genomes that can be consulted online [16]. This database is being used in genome sequencing projects as a collection for masking and annotation of repetitive DNA, because it contains a picture of the TE landscape in several genomes. However, most TE sequences from Repbase are prototypic consensus sequences of families and subfamilies of repeats. On the other hand, a specific TE database called Tefam presents TE sequences from three mosquito genomes: *An. gambiae*, *A. aegypti*, and *Culex quinquefasciatus *[17]. TEfam contains a more restricted number of TE families in the mosquito genomes.

Previous analyses of TEs in *An. gambiae *have identified several element families [3,18-24]; however, the data are often scattered in the databases or clearly unavailable.

The aim of this work was to describe the diversity of TEs within the *An. gambiae *genome following the use of the PILER tool, which identifies repetitive elements in whole genomes [25]--with a particular interest in the detection of new, active elements--as well as to consolidate a database in the form of a hyperlinked spreadsheet containing detailed and readily available information about the TE diversity and other repetitive elements found in the genome of *An. gambiae*. The purpose was to present the platform used in the identification and characterization of TEs in the genome of *An. gambiae*, which, in turn, can be used to screen other sequenced genomes.

## Results and Discussion

**Figure 1:**
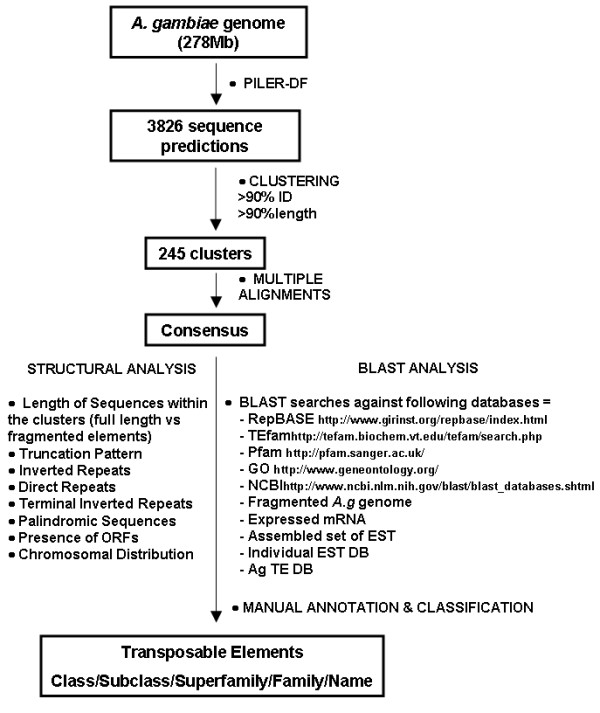
**Pipeline for the identification and characterization of transposable elements in the *Anopheles gambiae *genome**. Flow chart indicating the steps followed for the characterization of repeats in *An. gambiae*.

An important aspect in the study of the TEs in a genome is knowledge of their abundance and diversity, as well as classification and further organization of this information in a clear and comprehensive manner. Aiming at the discovery of unknown, active elements, we performed a search and characterization of TEs in the sequenced genome of *An. gambiae*. We used a combined strategy (Figure 1) to identify and characterize repetitive elements present in the genome of the primary vector of malaria, *An. gambiae*. Our strategy combines an extensive search of repetitive elements within this genome utilizing the PILER-DF algorithm as a first screening method, together with a detailed characterization of each of the retrieved sequences by analyzing signature characteristics of TEs as well as performing homology-based analysis of the obtained sequences to several databases. This automated pipeline includes the different steps suggested recently by Wicker, *et al*., [11] for classifying eukaryotic TEs. The results are compiled in a database of repetitive elements in the mosquito genome, called AnoTExcel (Additional Files 1 and 2) that provides information for all the TE families identified, along with offering the individual sequences found in each family.

### Database description

AnoTExcel is organized as an Excel spreadsheet with cells containing, in a hyperlinked format, the results obtained after the various analyses performed in the characterization of each family. Each line of the spreadsheet represents a cluster containing a variable number of sequences with a high degree of identity among them, constituting--in most of the cases--TE families. The spreadsheet columns contain the several analyses performed on the sequences and were grouped into four types, colored for easier viewing as follows: blue columns represent the classification of the families; green columns present the general characteristics of the sequences in clusters, such as presence of TIRs and their sequences, direct repeats (LTRs), consensus and centroid sequences, ORF sequences, *etc*. In orange columns are presented all the results of the blasts performed on several databases (see Methodology section), and in yellow columns (FZ-GQ) are results of new clustering performed by tblastx considering different degrees of identity over at least 50% of the length of the alignments (from 35% to 95% of identity on 50% of the length). This was performed to identify shorter fragments of sequences belonging to the same family that were included in different clusters when more stringent conditions were applied. Clusters that share some degree of identity are colored in column A in AnoTExcel.

This is not an exhaustive database, as some of the element families already known to be present in *An. gambiae *have not been identified by our algorithm; however, it has the important and distinctive characteristic of presenting all the individual sequences within each family (including fragments or short sequences belonging to TE families) as well as their global alignments in fastA format, the consensus and centroid sequences, and detailed information on their structural characteristics. In addition, it presents the results obtained after several blasts performed on different databases and includes in hyperlinked format the file with the significant matches to the given database.

This constitutes a rich resource that includes important information on the different families of TEs present in the genome of *An. gambiae *besides serving as a platform for the analysis of TEs present in other sequenced genomes.

AnoTExcel contains 3826 repetitive sequences larger than 400 nts in length that were grouped into 245 clusters (see Methodology section). The repetitive sequences are considered "intact" because they are globally alignable and "isolated" because they are surrounded by unique sequences; therefore, they correspond to insertional events of different repetitive sequences, mainly TEs [25]. Our strategy in generation of the clusters permitted the grouping of sequences belonging to the same TE family with different sizes or corresponding to different regions of the same element. Considering that the sequences identified by PILER-DF are isolated and unique, it is possible to state that those sequences represent different transposition states of the same TE family, allowing for evolutionary or dynamical analysis. The spreadsheet containing AnoTExcel can be found at http://exon.niaid.nih.gov/transcriptome/TE/A_gambiae/AnoTExcel-WEB.zip and the standalone data can be downloaded from http://exon.niaid.nih.gov/transcriptome/TE/A_gambiae/AnoTExcel-SA.zip

The latter file should be decompressed to the user's computer, and the Excel file should be opened from within Excel. AnoTExcel can be downloaded and stored in a personal computer; it occupies approximately 178 Mb and can be modified by adding or deleting columns according to the user's needs.

**Figure 2:**
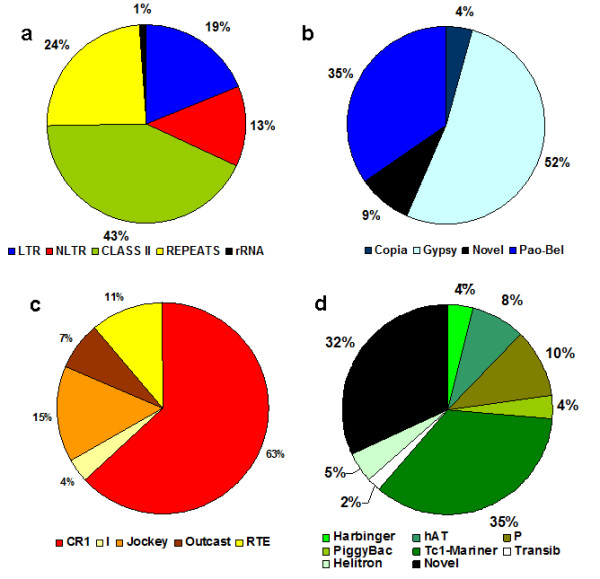
**Distribution of repeats in AnoTExcel**. (a) Distribution of clusters. (b) Distribution of long terminal repeat (LTR) superfamilies. (c) Distribution of non-LTR (NLTR) superfamilies. (d) Distribution of Class II superfamilies.

We were able to assign most of the retrieved sequences (75%) to a given TE class (I or II); the rest were classified as rRNA (1%), or repetitive sequences with no TE signatures (24%) and possibly expressed pseudogenes (Figure 2).

**Figure 3:**
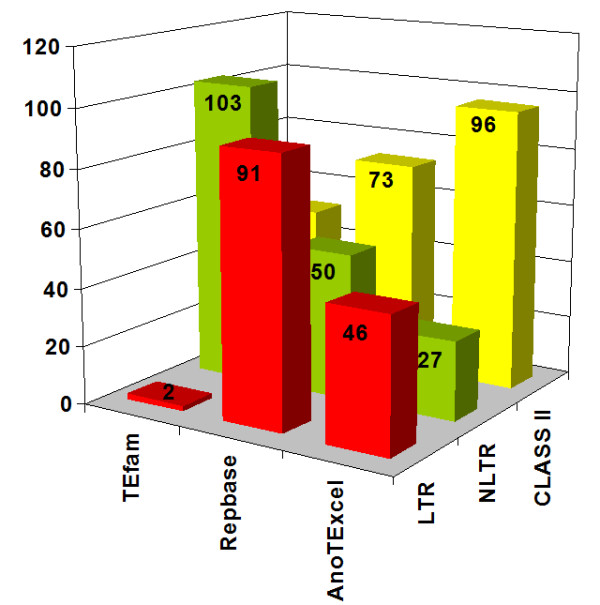
**TE distribution in Repbase, TEfam and AnoTExcel**. Number of LTR, NLTR and Class II families present in three databases: TEfam. Repbase and AnoTExcel. The numbers in the bars indicate the number of families in each category.

In order to assess the efficiency of our approach in identifying and characterizing TEs from the genome of *A. gambiae *we compared the number of sequences in AnoTExcel with those deposited in Repbase and TEfam. The results are shown in Figure 3. It should be noted that these figures are not 100% comparable since many elements are deposited in Repbase and TEfam under different names (overlapping). Also, Repbase and AnoTExcel present data on subfamilies (that might correspond to different transposition events in time but not to different families) that were included in Figure 3. In addition, some but not all of the elements present in TEfam are also deposited in Repbase (redundancy). We identified approximately half of the LTR elements that have been described so far in the genome of *An. gambiae*, few of the NLTRs and almost all the known Class II elements. Moreover, we were able to identify and characterize several families of elements (both Class I and II) that have not been reported previously, even using similar approaches for identifying TEs in the mosquito genome [20,24]. Further characterization of the TE families is given below.

### TE characterization

The TE families identified in AnoTExcel were classified according to their class, subclass, order, superfamily, and family, following the Wicker criteria for TE classification. We also classified the families according to the length of sequences in each cluster, *i.e*., according to the percentage of the cluster consensus sequence representing the full-length canonical element as: full-length (100% match), fragments or remnants (less than 10% of match) and, depending on the case, as Solo LTR (for solitaire LTRs resulting from the homologous recombination occurring in LTR elements), MITEs (miniature inverted terminal repeat elements), or Class II-NA (non-autonomous elements, already described as such in Repbase). The methodology used here permitted identification of families presenting both full-length elements and degenerate copies of the same family (mainly for Class I-NLTR elements). The LTR elements contain several full-length putative active sequences, while NLTRs and Class II elements are mainly constituted by fragments or remnant sequences. For the NLTR elements, the percentage of clusters considered as "full" in AnoTExcel is notably higher than the percentage of full-length sequences that in fact exists, indicating that many of the NLTR clusters constitute a mixture of both full-length and fragmented sequences.

Notably, although the *An. gambiae *genome has been scrutinized in search of TEs before using several methodologies [22-24,26], we have been able to identify novel TE families that have not been previously identified, as detailed further below.

### Elements Class I, order LTR

The LTR elements in the genome of *An. gambiae *constitute a numerous order, although there are only three superfamilies: Ty1-Copia, Pao-Bel, and T3-Gyspsy, the last being the most diverse LTR superfamily within the mosquito genome.

The LTR elements identified in AnoTExcel were characterized based on the presence of flanking LTRs and/or based on positive matches to Peptidase_A17, RVT_1 or 2 or RVE in Pfam or directly due to their significant matches to known LTR elements already deposited in the TE databases (TEfam and Repbase) or in the GenBank non-redundant database.

In AnoTExcel, they correspond to 19% of all the families identified (Figure 2), and 26,4% of the total TEs identified, totalizing 46 different clusters, and are represented by members of the main three superfamilies. Novel elements corresponding to the Copia and Pao-Bel superfamilies were also identified and will be described later.

Full-length and fragmented elements are not equally represented in the different LTR superfamilies. While the majority of the Pao-Bel elements are represented by full-length copies (68%), the Gypsys are represented by Solo-LTRs (50%), and the few Copia families identified correspond to full-length elements.

The predicted activity of the full-length sequences was also studied (Additional File 3). Based on *i) *the degree of nucleotide identity among the sequences belonging to the same families; *ii) *the degree of identity among the LTRs of individual elements (identical LTRs in all of them); *iii) *the indication of expression based on positive matches to EST databases in AnoTExcel; and *iv) *the presence of full-length ORFs containing conserved domains for the proteins involved in transposition, we concluded that a substantial fraction of the LTRs described here corresponds to putatively active elements, as indicated by their presence within cDNA libraries (clusters 45, 115, 238, 140, 130, 191, 110, 199, 101, 98, 131, 182, 104, 119, 171) (Additional File 3). The LTR families contain elements presenting an average nucleotide identity higher than 99.5%, and comparison of the LTRs among all the sequences in each of the clusters showed a high degree of identity for the three superfamilies (average of 99.13% for Pao-Bel, 99.04% for Copia, and 99.11% for Gypsy). In addition, the identity between the two LTRs present in the individual sequences was calculated and appeared to be very high for some of the LTR members (averages of 99.81; 99.64, and 99.83% for Pao-Bel, Copia, and Gypsy, respectively) (Additional File 3). The presence of full-length ORFs in the sequences reinforces the idea that these families are active or have been very recently inactivated [19].

### Copia elements

The Copia superfamily in the mosquito genome is represented by five different families: Copia1-5_AG [27-29] and the Mtanga family [30]. These families have been reported to Repbase, and none of them is present in TEfam. In AnoTExcel, elements belonging to two families, Copia3_AG (cluster 172) and Copia5_AG (cluster 150) were identified. The consensus sequence of these two families is identical to the corresponding consensus sequences deposited in Repbase. Element Copia3_AG is apparently a truncated element with no conserved protein domains. The Copia5_AG sequences present some characteristics of activity, such as the presence of conserved protein domains for RT and RVE and identical within sequence LTRs, even if they presented no positive matches to the mRNA and the EST databases (Additional File 3).

### Novel Copia elements

**Figure 4:**
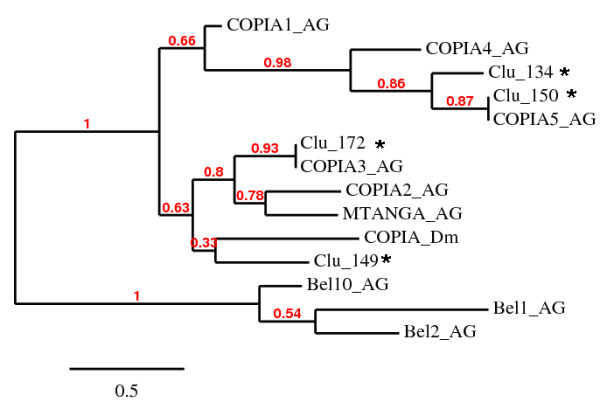
**Maximum likelihood phylogenetic tree of the pol region of Copia elements from the genome of *Anopheles gambiae***. The final alignment encompasses a region of 1928 aa positions. The tree was generated using default settings for MUSCLE 3.7 (as alignment tool), Gblocks 0.91 b for alignment refinement, PhyML for tree generation, and TreeDyn for tree drawing as implemented in [69]. Red numbers above the branches indicate a confidence index based on the approximate likelihood-ratio test (aLRT) for branches [72]. An * indicates sequences identified in AnoTExcel. Three sequences belonging to the Pao-Bel superfamily were used as outliers for tree construction.

Two clusters (numbers 134 and 149 of AnoTExcel) were characterized as elements belonging to the Copia superfamily, although it was not possible to assign them to any of the already described families within this superfamily since they present high nucleotide distances against all the previous characterized Copia elements (Additional File 4). The consensus sequences of these clusters showed significant matches by tblastx with the Copia5_AG consensus sequence and Copia_DM from *Drosophila melanogaster *[31], respectively (see AnoTExcel). A phylogenetic analysis including all Copia elements previously identified in *An. gambiae *and a Copia element from *D. melanogaster *showed no clear clustering of any of these sequences with the previously described Copia elements (Figure 4).

Additional File 5 presents the main characteristics of the sequences belonging to the novel LTR elements described in AnoTExcel. The four sequences within cluster 134 are 4399 long with a p-distance at the nucleotide level, considering the full-length alignment of 0.0009 (standard deviation [sd] = 0.0003). The LTRs are 149 nts long and present a very high degree of identity (p-dist = 0.0033; ds = 0.0038). In two of these sequences, the 3' and 5' LTRs are identical (Additional File 5), indicating that they have inserted recently and have not had time to accumulate mutations between the LTRs. The consensus sequence presents an ORF of 1350 aa containing conserved regions for integrase (RVE), reverse transcriptase (RVT_2), and RnaseH, a primer-binding site (PBS) for proline, and a polypurine signal at position 4234, information that reinforces the idea of activity of this family.

The mean aa distance of the consensus sequence of clusters 134 to each of the consensus of the already described Copia elements shown in Figure 4 is 0.6520, indicating that they do not correspond to any of the already described Copia families (Additional File 4). The consensus sequence of cluster 134 shows two regions spanning 2008 and 1560 nts with 65% and 69% identity, respectively, with element Copia-5_AG. The unique ORF of cluster 134 presents 56% identity along the whole sequence with Copia5_AG. Still, the 149-nts-long LTRs of cluster 134 do not show any identity with the 108-nts-long LTRs from Copia-5_AG, indicating that they are not members of the same TE family.

The synonymous substitutions among the sequences in cluster 134 were more than six times more frequent than the substitutions at non-synonymous sites, which indicate the presence of purifying selection operating on them. Tajima's test, on the other hand, was not significant for these sequences, although the fact of being four sequences with few segregating sites along the alignment might influence the ability of the test to detect selective pressures operating on them (Additional File 5).

The consensus of the sequences in this cluster has been deposited in Repbase under the denomination Copia-6_AG.

The four sequences in cluster 149 are 2245 nts long with a mean p-distance of 0.0083 and sd = 0.0014, considering the whole alignment. The LTRs are 168 nts long and are identical in all the sequences, suggesting a recent transposition event.

The consensus sequence has an ORF of 615 aa that contains no conserved regions. The sequences present two PBSs, three sequences for valine, and one for methionine. The consensus sequence for this cluster shows no regions of identity with previously identified elements. In the phylogenetic analysis shown in Figure 4, the cluster 149 sequence groups with a Copia element from *D. melanogaster*, although with a non-significant bootstrap value.

The mean aa distance of the consensus sequence of cluster 149 to each consensus of the already described Copia elements shown in Figure 4 was 0.7645.

The blasts performed on an EST library as well as on a predicted expressed mRNA, both from *An. gambiae*, gave positive matches (Additional File 5). So, even if this sequence apparently corresponds to an inactive element, it is being expressed, as demonstrated by its positive match both to the mRNA and the EST databases, which indicates the activity of this family. The consensus of the sequences in this cluster has been deposited in Repbase under the denomination Copia-7_AG.

### Superfamily Pao-Bel

According to Repbase, there are 20 families of Pao-Bel elements in the mosquito genome (AGM1 and Bel1-19_AG). AnoTExcel presents sequences of 15 of them plus 2 novel families. Eleven of these clusters contain full-length sequences that were further analyzed. They all contain ORFs with protein domains for RVT, Pep17, and RVE (with the exception of cluster 110, belonging to the family Bel16_AG, which does not present RVE domain), contain very high sequence identity within each cluster and, in all the cases, the within-element LTRs are identical, indicating that they are active at present or were so very recently. In addition, ten of these families are being expressed, as indicated by their positive matches to the EST database (Additional File 3). A phylogenetic analysis of the RT-PeptA17 conserved domains of the Pao-Bel elements confirmed the classification of the clusters based on the information present in AnoTExcel. In this phylogeny, only those sequences containing conserved domains for the RT were included.

### Novel Pao-Bel elements description

Two families of elements characterized as Pao-Bel due to their matches to Pao-Bel elements deposited in Repbase by tblastx (clusters 174 and 185, respectively) were further characterized.

Cluster 174 is composed of three sequences presenting a high degree of identity among them (p-dist = 0.0068; SD = 0.0010) (Additional File 5) and a total length of 5769 nts; two of them contain 213-nts-long identical LTRs, indicating that these sequences might have transposed very recently. The consensus sequence presents an ORF of 1758 aa with conserved domains for RT, PeptA17, and RVE, also indicating that sequences might belong to an active or recently active family. The blastn performed on TEfam and Repbase showed that this family has not been reported before; still, the tblastx on Repbase gave positive matches to several Pao-Bel families, indicating that this family belongs to the Pao-Bel superfamily.

**Figure 5:**
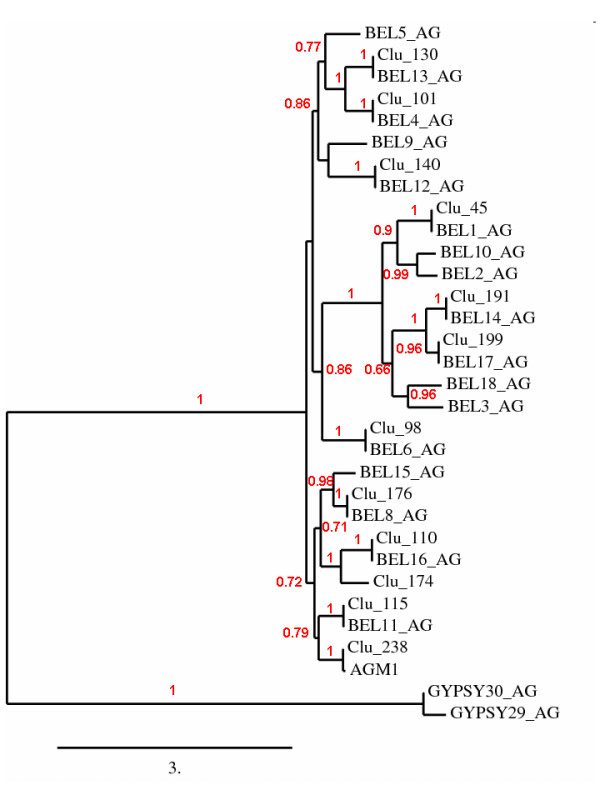
**Maximum likelihood phylogenetic tree of the pol region of Bel elements from the genome of *Anopheles gambiae***. The final alignment encompasses a region of 423 aa positions. Refer to legend of Figure 3 for detailed information. Two sequences belonging to the Gypsy superfamily were used as outliers for tree construction.

The consensus sequence of this cluster has three regions (nucleotide positions 700-1132, 2538-3907, and 4440-5357) with 63% identity with element Bel16-I_AG. Phylogenetic analysis performed with already characterized Pao-Bel elements showed that the consensus sequence of cluster 174 grouped together with the representative sequence of BEL16_AG with a significant likelihood branch support value (Figure 5). Nonetheless, the LTRs of both families show no identities and present different sizes, indicating that they do not correspond to the same family.

The sequences in cluster 174 present 100% identity with two cDNA spanning 786 nts from a cDNA library, indicating that this elements is being expressed.

On the other hand, we identified more substitutions in synonymous than in non-synonymous positions, with a dN/dS value of 0.2093, indicating a selection pressure on the ORF of these sequence. Tajima's test was not executed due the small number of sequences.

The consensus of the sequences in this cluster has been deposited in Repbase under the denomination Bel20_AG.

The three sequences in cluster 185 are 3803 nts long, with a p-distance of 0.0033 and sd = 0.00077, presenting LTRs of 227 nts that are identical within each element. The consensus sequence presents an ORF of 927 aa that has no conserved domains for known proteins. The LTR finder program detected a PBS for asparagine and a PPT signal.

The results obtained after blastn in the TE databases, TEfam and Repbase, indicate no identity with known elements; nevertheless, the tblastx against Repbase shows significant e-values with several Pao-Bel elements, mainly with families Bel-18_AG [32] and Bel-3_AG [33]. The LTRs of these families are not, however, similar in length or nucleotide composition to the LTRs of cluster 185.

The blasts performed on an EST library as well as on a predicted expressed mRNA, both from *An. gambiae*, gave positive matches (Additional File 5). Nevertheless, the absence of conserved domains in the ORF present in this sequence made it impossible to compare it phylogenetically with other Pao-Bel sequences.

Analysis of dS vs. dN showed no differences in the proportion of substitutions in synonymous vs. non-synonymous positions, indicating a neutral evolution (Additional File 5). The general characteristics of the sequences in this family suggest that it corresponds to a non-autonomous family composed of few elements. The consensus of the sequences in this cluster has been deposited in Repbase under the denomination Bel-21_AG.

### Superfamily Gypsy

The Gypsy superfamily is the most diverse LTR superfamily in terms of the number of different families that have been previously identified [16,34]. Traditionally, this superfamily had been classified into nine different lineages based on phylogenetic analysis of the RT, RnaseH, and INT domains [35]. Six of these lineages have been previously reported in insects and five of them (Gypsy, Mag, Mdg1, CsRn1, and Mdg3) were described in *An. gambiae*. Tubio, *et al*., reported the identification of a huge variety of families within each of these lineages.

In AnoTExcel, we identified families with full-length sequences belonging to each of the so-called lineages, but we failed to identify the majority of the individual families.

AnoTExcel presents six clusters with full-length sequences in addition to other clusters presenting fragmented elements or solo LTRs. The six full-length clusters have signs of activity (Additional File 3), presenting functional ORFs, and five of them have positive matches to the EST library.

Most of the Gypsys that we identified correspond to Solo elements. In *An. gambiae*, Tubio *et al*. found a high proportion of solo LTRs belonging to this superfamily [19] and interpreted this fact as a slow turnover of LTR elements from the genome of *An. gambiae*. This would mean that each individual copy remains for a longer time in the genome, enhancing the chance of homologous recombination to occur, which is the proposed mechanism to generate this type of deteriorated LTR [36-38]. The lower proportion of Solo LTR elements in the other LTR families might suggest that they are not equally present in all LTR elements.

### Elements Class I, order non-LTR

The non-LTR elements identified previously in the *Anopheles *genome and deposited in TEfam and/or Repbase constitute a very diverse order of elements composed of 22 different superfamilies, 7 of which were identified in AnoTExcel, belonging to the superfamilies RTE, Outcast, Jockey, I, and the majority of them to the CR1 superfamily. Some families were considered as remnants of NLTR elements for presenting little identity with NLTR elements from TEfam and/or Repbase by tblastx.

The NLTRs are usually between 3 and 8 Kb long, and they usually contain two genes: the pol gene, fundamental for their replication, and the gag gene, which it is also present in the LTR retrotransposons and retroviruses.

In AnoTExcel, the NLTR elements were classified based on their positive matches to RVT_1 in Pfam and/or their positive matches to polyprotein in the TE databases, as well as positive matches to sequences present in the specific TE databases or GenBank NR database.

In total, 32 clusters were classified as NLTRs (Figure 2), corresponding to 13% of all the families identified and 18,4% of the total TE families. The families of NLTR elements contain, overall, more numerous sequences than the LTRs (20.6 versus. 5.1 sequences per family); this is to say, each family is more abundant and more heterogeneous, because most of the clusters are composed of both full-length and fragmented sequences. Most of the full-length sequences present conserved protein domains for exo-endo phosphatase and RT (Additional File 6). Nine of these clusters present a second ORF but no apparent conserved domains. The p-distances among all the sequences within each cluster are small and, together with the significant results of Tajima's test as well as the significant matches to the EST library (Additional File 6), indicate that some of these clusters are transcribed.

The majority of the NLTR families present in AnoTExcel correspond to the CR1 superfamily. Most correspond to fragmented sequences, and the full-length sequences keep a high nucleotide identity.

AnoTExcel also presents one cluster with signs of expression belonging to the I superfamily. Both the Jockey and Outcast superfamilies seem to have active members that have already been reported [18]. Three clusters belong to the *RTE *superfamily, two of them present full-length ORFs and positive matches to the EST library, and one has a positive match to the predicted expressed mRNA.

### Class II

The actual transposons or Class II elements are characterized by the presence of a gene coding for a transposase enzyme flanked by TIRs and have been recently classified into two subclasses (1 and 2) according to the number of DNA strands that are cut during the transposition event [11]. Elements of class II belonging to subclass 1 and presenting TIRs have been subsequently classified into nine superfamilies, six of which have been previously identified in the *Anopheles *genome. Members belonging to all these superfamilies are present in AnoTExcel, where class II elements have been classified based according to the presence of TIRs and on the positive matches to already characterized elements deposited in any of the databases analyzed.

The majority of all the TE families identified in AnoTExcel belong to this class (43%), (Figure 2a), which corresponds to 55.2% of all the TE sequences retrieved by PILER. Elements belonging to six different superfamilies (P, Tc1-Mariner, Transib, PIF-Harbinger, piggyBac, and hAT) were identified, as well as Helitrons that belong to subclass 2. Tc1-Mariner constitutes the most numerous superfamily, with 39 different families, representing 35% of the class II families (Figure 2d).

We were not able to identify copies of full-length piggyBac families [39] or the Herves element, which correspond to a class II active element [40]. This element belongs to the hAT superfamily, and although we identified other hAT elements in the genome, all of them constitute truncated copies [40]. On the other hand, 32 clusters with a variable number of sequences harboring TIRs with no relationship to previously known elements were also identified. These elements have been classified as novel Class II MITE-like elements and will be later characterized.

The great majority of the class II elements identified here correspond to highly deteriorated sequences, represented by elements with different degrees of deterioration, including several families already characterized of MITEs, NA (non-autonomous families already identified in Repbase), fragments, and a few remnant clusters. Only four clusters harbor full-length sequences, belonging to superfamilies Tc1-Mariner (clusters 41, 114 and 133) and P (cluster 161) (Additional File 7). All contain full-length TIRs and, except for cluster 114, they contain conserved domains for transposase and positive matches to the EST library, so they probably constitute active or recently active elements (Additional File 7).

### MITEs

**Figure 6:**
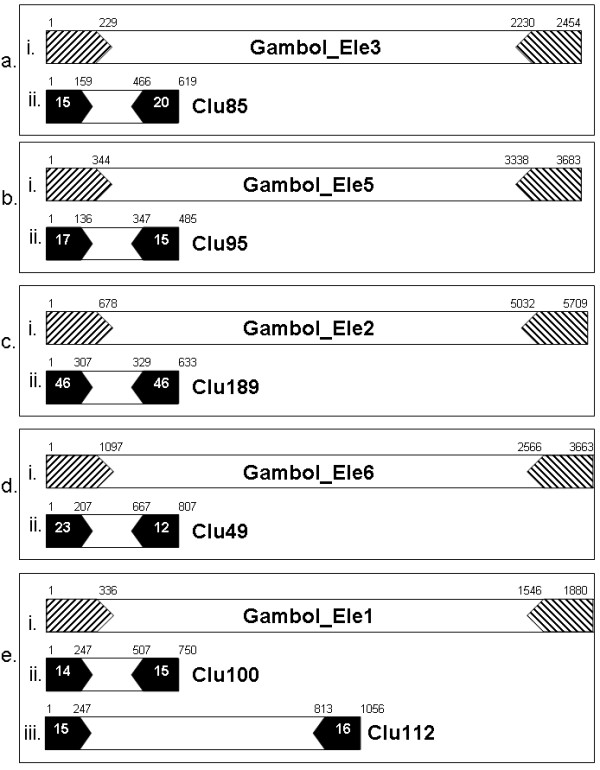
**Structure of MITE-like elements belonging to the Gambol superfamily**. (i) The diagram represents the Gambol element as described in Tefam [17]; striped arrows represent the terminal inverted repeats (TIRs). (ii) Representation of the structure of the MITE-like elements. Black arrows represent TIRs with the number of mismatches with the TIRs from the complete elements in white. The numbers above the diagrams are the nucleotide positions where the TIRs begin and end in each case. In each box, a-e. represent the original Gambol element (above) and the MITE-like element from AnoTExcel.

These elements were originally described in plants [41-43] and have been found in other eukaryotic organisms, including mosquitoes [44-46]. They are small, non-autonomous elements (~100-500 bp) that contain TIRs but do not codify for any protein. They are believed to originate from full-length active elements that lose their coding capacity but maintain the TIRs, which allow their mobilization, in a parasitic manner, by active transposases. There is ample evidence indicating a relationship between active elements and MITEs, although there is no clear mechanism that explains their generation [47-52]. They are normally present in high copy number. In AnoTExcel, 18 clusters were classified as MITEs of previously characterized families. Some of them had been identified as MITEs before, belonging to the superfamilies Tc1-Mariner, P, and Harbinger, while others have not been reported as such until now (*e.g*., the MITEs from the Gambol elements). The Gambol elements belong to the Tc1-Mariner superfamily; they contain the characteristic DD34E motif [53] and are represented by 13 different families that are deposited in TEfam. Here we identified ten families related to Gambol elements, six of which have TIRs with high identity with the TIRs of the original elements but smaller size (Figure 6a-e). This might indicate that just a part of the TIR is necessary for element mobilization. In the six examples shown here, the extreme outer region of the TIRs is maintained while the inner region of the TIRs is not present. Also, the internal region of all the Gambol MITE-like elements presented in Figure 6 have no significant nucleotide similarity with any internal region of the Gambol counterpart elements. They all have quite small sizes, and in the case of the MITE-like elements of Gambol_Ele1, cluster 100 and 112, they constitute two subfamilies with almost identical TIRs but with different sizes and different internal regions in nucleotide composition, indicating that they probably originated in different events (Figure 6e).

It is possible that more than one family of MITEs evolved from a unique master copy, generating diverse families that keep on transposing and evolving depending on the presence of an active element in the genome, as has been demonstrated for P elements in the *An. gambiae *genome [51]. The origin of the internal region of MITEs is controversial: it might derive from internal regions of the respective master TEs followed by nucleotide degeneration or, alternatively, it might originate from an ectopic site [51].

### MITE-like novel elements

32 clusters presenting TIRs or palindromic flanking ends, but with no identity to known TEs, either in the TIR or in the internal region, were identified. TIR lengths vary from 18 to 204 nts and their total length from 430 to 1678 nts, and six of them are, in fact, palindromic repeats. Palindromic TEs have been previously identified in *Caenorhabditis elegans *[54] and *D. melanogaster *and also in Mariner elements identified in *An. gambiae *[55]. Palindromes are predicted to form secondary structures; in bacteria, repetitive extragenic palindromic elements (REP) have been described as hotspots for transposition, indicating a relationship between REPs and TEs [56]. Little is known about the role of these sequences within genomes, however.

Nine of these clusters constitute sequences longer than 1000 nts, and 17 are longer than 800 nts (Additional File 8). Some of these families present more than 60 individual copies, but none showed signs of autonomous activity, as they only present small ORFs and no signs of conserved motifs. A significant proportion presents matches to EST databases, suggesting that they are being expressed, and five of them present significant matches to the mRNA databases.

Tajima's test for the group of sequences within these clusters showed significant values in 14 of them. Tajima's D is normally used as a selective neutrality test statistic; however, it has been suggested that sudden population expansions can lead to negative D values, moving the observed D value outside the 95% confidence interval derived for a neutral locus and stationary population [57]. Considering this, it is possible that the significantly negative D values obtained for the clusters analyzed here correspond to TE families that are in a process of expansion--indeed, they all constitute quite numerous families within the *Anopheles *genome.

We believe that these sequences are MITE-like elements, although we cannot rule out the putative master TE due to the lack of identity with known elements.

**Figure 7:**
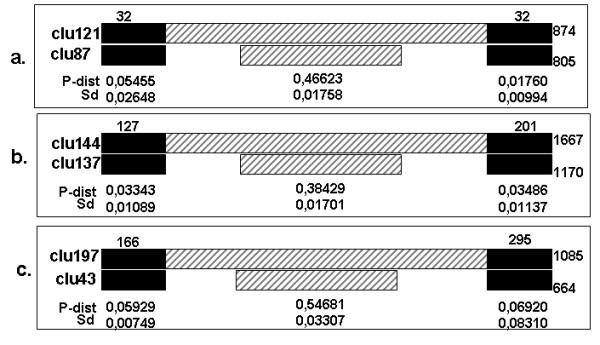
**Structure of three MITE-like families belonging to unknown transposable elements**. (a) Family of MITE-like elements including clusters 121 and 87 from AnoTExcel. (b) Clusters 144 and 137; and (c) clusters 197 and 43. Black squares represent the terminal inverted repeats, and the numbers above them are the lengths of the identity region. The internal regions are represented by striped rectangles. The numbers at the end of the diagrams show the length of the elements in nucleotides. The numbers behind these squares represent the p-distance and the standard deviations for the homology regions of the sequences in the alignments.

MITEs are known to be present in several genomes, and they have been associated with master TEs [51]. They share their TIRs with active elements, and it appears that they manage to survive and spread within genomes by borrowing the transposase from active elements. What is not known is whether their internal regions derive from internal regions of the master elements or if they are copied from an ectopic site by a conversion process after a double-strand break. The clustering with less stringent conditions performed by tblastx, shown in the last columns of AnoTExcel, permitted the identification of sequences with a high degree of identity only among the TIR regions, *i.e*., different subfamilies of MITE-like elements. Three groups of clusters among the novel MITE-like (clusters 121-87; 137-144; and 43-197) were identified by the less stringent clustering. In all cases, they share high identity in TIR regions but low identity in internal regions (Figure 7). It is possible that the diverging time between these elements is so long that it is impossible to find any detectable similarity.

It is interesting to note that many known MITEs, such as the Stowaway family in rice, have no homologies with other TEs, leaving an open question regarding the origin and means of replication of these small, non-autonomous elements [58]. This appears to be the case for the orphan MITE-like sequences presented here.

It is apparent that the Class II elements present in the genome of *An. gambiae *are composed of a variety of different structurally degenerated sequences that might represent different stages in the process of deterioration of these elements, which in turn might be differentially involved in the regulation of Class II families [59].

## Conclusions

Two basic approaches for the task of repetitive sequence identification at the genome scale are commonly used; they are based either on the similarities of a query sequence to already described elements (homology strategies) or on recognition of TEs' intrinsic characteristics, such as their repetitive nature within the genome or the presence of structural motifs such as LTRs, TIRs, or specific target site duplications (TSD) in a query sequence (homology-independent methods) that are specific of certain families of TEs.

Here, we used a combined strategy to identify and characterize the repetitive elements present in the genome of the primary vector of malaria, *An. gambiae*, and to present all the gathered information in an easy and accessible manner. We described several families of elements present in the mosquito genome, as well as novel elements that have not been described before.

Previous works [24,60] have shown that use of multiple softwares increases the detection of TEs and that the use of PILER alone has a low performance for the detection of TEs in a genome. It is worth noting that although our approach missed the detection of some TE families, it permitted the identification of novel TE families as well as MITE-like elements from previously described Class II families that had not been described before. This is related with a unique feature of the PILER program in that it finds and distinguishes different classes of repeats by their characteristic features, implementing different search methods for dispersed families (PILER-DF), tandem arrays (PILER-TA), pseudo-satellites (PILER-PS) and terminal repeats (PILER-TR). The ability of PILER to incorporate biologically informed constraints into the repeat discovery process allows the identification of repeats that are more likely to be TEs and, in many cases, of unknown TE families or subfamilies. Also, it facilitates the identification of fragmented or deteriorated sequences of a given family, which in turn gives a broader idea of the structural diversity within the TE landscape of the genome. It has also been shown previously (72) that the consensus sequences constructed by PILER are longer than those produced by other similar methods, such as RepeatScout or RepeatFinder and the composition of the repeat libraries generated are similar than the ones obtained by these other programs (72). On the other hand, PILER suffers from certain limitations including the inability to recognize TE families with few members and a relatively low representation of NLTRs in its output file which has been previously recognized (72) with a concomitant higher representation of Class II and LTRs. This fact has been related to the higher identities that LTR and Class II elements show in their flanking sequences.

AnoTExcel is a platform for analysis of the repetitive elements present in *An. gambiae *that contain TEs families with different degrees of deterioration and which include the results of several analyses, allowing its use by researchers interested in several areas of TE biology such as variability of terminal regions (TIRs or LTRs) and deterioration processes. This compiled information can serve as a starting point for future investigations of TE families in *Anopheles *or in different species. The platform used to identify and characterize TEs in the mosquito genome can be easily used to generate analogue databases in other sequenced genomes, which can be useful for understanding the dynamics of TE families evolving in different genomic contexts.

This platform can also serve for analysis of TEs in sequenced genomes of other Anophelines, allowing for comparative purposes. This could permit the study of the dynamics of TEs in different genomic environments, including the understanding of the process of deterioration of certain TE families.

## Methods

### Pipeline description

The program PILER-DF [25] was used to identify repetitive sequences within the genome of *An. gambiae *(release 37, February/2006). PILER is designed for identification of repeats in an assembled genomic region. This program searches for all the repetitive sequences in a given genome. For doing so, it uses the PALS algorithm (Pairwise Aligner for Long Sequences) that generates an alignment of the genome to itself in order to find local alignments. PALS records the coordinates (i.e. start and end points) of each substring that shares significant sequence similarity (>94%) with at least one other substring. Overlapping substrings are condensed into 'piles' (_elements) and piles sharing significant sequence similarity are grouped into families and further into superfamilies, which contain sequences that are locally alignable (this corresponds eventually to different fragmented elements belonging to the same family). A consensus sequence is generated for each family. The program was run under the default settings; consequently, intact, isolated copies of repeats that appear three or more times in the genome and are larger than 400 bp were obtained. We were interested in the analysis of abundance and diversity of repetitive elements as well as in TE dynamics; we therefore analyzed and incorporated into the database all the hits obtained by the PILER algorithm.

Subsequently, all the sequences were clustered into groups of elements sharing more than 90% identity over more than 90% of the length of the sequence. TEs belonging to the same family are expected to be present within these clusters. To discover identities among the already grouped clusters, all the obtained repeats were clustered considering different degrees of identity (35, 50, 75, and 90%) over more than 50% of the sequences length (See columns FY to GP of AnoTExcel).

### Multiple alignments

All the sequences within each cluster were subsequently aligned using MUSCLER (see column AL in AnoTExcel), an algorithm developed by Dr. J. M. C. Ribeiro, which aligns the sequences progressively using MUSCLE [61]. Briefly, the two longest sequences are first aligned, and shorter sequences are progressively added onto the previous alignment. This approach was utilized because the alignment of sequences of very different lengths and belonging to different regions of the same gene that were present in certain clusters in the dataset was quite complicated with programs such as CLUSTAL [62] or MUSCLE alone. The program MUSCLER calls MUSCLE to run the alignments using the following parameters: center = -1; gapopen = -500; and gapextend = -50. Visual inspection of the alignments using the program MEGA1.4 [63] helped in refinement of the final alignments.

### Consensus and centroid sequences

Consensus sequences were obtained from the multiple alignments for each of the clusters. For drawing the consensus sequence, we retained in each position of the sequences the nucleotide that was present in more than 50% of the sequences, independently of the number of sequences spanning that position. In this way, we were assured of having the longest possible consensus sequence. Those positions represented by less than 50% of the sequences in the whole alignment are indicated in small letters in the consensus sequence.

We also calculated the centroid sequence for each cluster and compared it to the consensus. The centroid sequences were obtained by choosing the sequence within each cluster that obtained the highest sum of scored value when summing all the scores of all-to-all blasts performed among all the sequences within each cluster.

As a means of corroborating how representative the consensus sequence was, the centroid and consensus sequences were blasted against each other, and the length of the consensus/centroid sequences were compared (results presented in Column J in AnoTExcel).

Even if--in a substantial number of clusters--the difference between the largest and the shortest sequences was important, we were still assured that the size of the consensus sequence was the largest possible for each cluster.

### Detection of ORFs

The presence of ORFs was deduced from the six frames of the nucleotide sequences belonging to the consensus sequence for each cluster (Columns BA, BB, and BC of AnoTExcel). Consensus sequences were also blasted against a bank of TEs or ORFs of repeats that was compiled based on the TE-specific databases available, Repbase [16] and TEfam [17].

### Detection of protein conserved domains

The software "Conserved Domains" from the National Center of Biological Information (NCBI) [64]http://www.ncbi.nlm.nih.gov/Structure/cdd/wrpsb.cgi was used for detection of conserved domains in the predicted ORFs of the TEs.

### TE signatures: inverted repeats (IR), direct repeats (LTR), and palindromic sequences

A blast of each sequence against *i) *itself and *ii) *its complementary reverse using the program Blast 2 Sequences [65] was used to detect inverted repeats (IRs), direct repeats (LTRs), and palindromes within the sequences. IRs were further classified as terminal (TIR) if they were present in the first 10% and in the last 90% nucleotides of each sequence; otherwise they were considered sub-terminal. The program LTR-finder [66] was used to help in the identification of novel LTR elements in the genome.

### Blast

Blastn was used to compare each of the repetitive element consensus sequences to the following databases: *i) *TEfam; *ii) *Repbase; *iii) *the collection of repetitive elements from the genome of *An. gambiae *described by Smith, *et al*., in 2007 [20,24]; *iv) *the annotated Gene Ontology (GO) databases [67]; *v) *the non-redundant (nr) protein database from GenBank; *vi) *the nr nucleotide database from NCBI; *vii) *an assembled set of 180,000 ESTs from *An. gambiae*; *viii) *an individual EST database; *ix) *pfam [68]; *x) *a gag protein database; *xi) *a transposase database; and *xii) *a fragmented *An. gambiae *genome database. A tblastx search was performed against the TEfam and Repbase databases to facilitate detection of highly divergent or deteriorated elements. Hits with e-values smaller than 10^-15 ^were considered positive for annotation purposes.

### Phylogenetic analysis

To characterize the novel TE elements here described, we performed phylogenetic analysis with previously described elements belonging to the same TE order, obtained from Repbase. We used a pipeline for phylogenetic analysis based on the MUSCLE algorithm for sequence alignment, a maximum likelihood algorithm PhyML for tree building, and TreeDyn for tree rendering with default settings [61,69-73].

### Annotation of sequences

Clusters were manually annotated based on the similarities of the consensus sequences to repetitive elements from curated libraries specific for TEs (TEfam and Repbase) as well as on the presence of specific TE signatures *(i.e*., presence of LTRs, IRs, or matches to proteins of retroviral or TE origin). Their matches to the other databases also helped in the annotation process. Repetitive elements were classified by class, subclass, order, superfamily, family, and element type for each cluster.

### Database construction

A database (AnoTExcel) with the information collected for each of the repetitive element families is presented in the form of an Excel spreadsheet containing hyperlinked cells to the different results obtained after the analysis performed on the sequences. The different rows in the spreadsheet (a total of 245) represent each of the clusters, which are composed of a variable number of sequences (ranging from 3 to 250). The columns present both structural (headings in green in AnoTExcel) as well as homology-based (headings in red) information as follows: the first column contains the cluster identification number hyperlinked to the consensus sequence obtained for that cluster (Column A); the next six columns (headings in blue) contain information regarding identification of the TEs. Structural analyses of the consensus sequences include analysis of the presence of ORFs (three columns including the size of the larger ORF in nucleotides, the amino acid (aa) sequence of the ORF, and the frame where it was found) and of the length of sequences within each cluster (four columns show the nucleotide [nt] length of the longest, shortest, and consensus sequences as well as the longest/shortest sequence ratio for each cluster.

An analysis to determine whether there is a pattern to the terminal deletions in sequences of certain families was also performed. Analysis of the 5' or 3' truncation was performed, and the number of gaps "-" at the 5' and 3' of each sequence within the alignments were counted. In this way, the 3' or 5' deletions were calculated and a graphical description based on these results created. The results, hyperlinked in the column named "Mean average length of sequence with gaps and link to truncation analysis" shows a representation of the alignment, as a .txt file, that indicates the deletions present at the extremes of the sequences within a given cluster. The fraction of sequences with IRs belonging to terminal, palindromic, direct, or other IR is also presented in different columns of the spreadsheet as well as the presence of LTRs. All the IRs present in the sequences of the same cluster were aligned, and a consensus sequence of the repeats was drawn. Alignments of the repeats found within the sequences are presented as an .aln file and are hyperlinked to their respective cell. Finally, the multiple alignments of the sequences performed with Clustal and MUSCLER are hyperlinked to the respective columns (in fastA and html formats). The spreadsheet also contains homology-based results to curated databases. Blast matches were considered significant when their e-value was lower than 10^-15^. The best matches to PFAM and to custom-made TE-specific databases (columns BF to BR), including gag-specific and transposases subsets that are hyperlinked, were also considered.

For each blast search performed, the characteristics of the matches are presented as different columns in the spreadsheet: 1) e-value, 2) best match, 3) score, 4) extent of the match, 5) length of best match, 6) % identity of the match, 7) % match length, 8) first residue of match, 9) first residue of sequence, 10) number of segments, and 11) orientation of output.

## Abbreviations

aa: amino acids; *an. Gambiae: Anopheles gambiae*; EST: Expression Sequence Tags; Gb: giga bases; GO: Gene Ontology; IR: Inverted Repeats; LTR: Long Terminal Repeat; mb: mega base; MITE: Miniature Inverted Terminal Element; NCBI: National Center for Biological Information; NLTR: Non-LTR; nr: non-redundant; nts: nucleotides; ORF: Open Reading Frame; PBS: Primer Binding Site; Pep17: Peptidase-A17; REP: Repetitive extragenic palindromic element; rRNA: ribosomal RNA; RT: Reverse Transciption; RVE: Integrase; RVT: Reverse Transcriptase; TE: Transposable Elements; TIR: Terminal Inverted Repeats; TSD: Terminal Site Duplication.

## Competing interests

The authors declare that they have no competing interests.

## Authors' contributions

RDFM, CJS, and JMCR helped analyze the data; CJS assembled the PILER pipeline; JMCR assembled the PILER results into spreadsheets; RDFM analyze the data and wrote the bulk of the manuscript; CJS and JMCR helped write the manuscript. All authors have read and approved the final manuscript.

## Supplementary Material

Additional file 1 **AnoTExcel database in standalone format**. It includes the links that need to be extracted to the user's computer http://exon.niaid.nih.gov/transcriptome/TE/A_gambiae/AnoTExcel-SA.zip. Click here for file

Additional file 2 **Web-based links of AnoTExcel**. http://exon.niaid.nih.gov/transcriptome/TE/A_gambiae/AnoTExcel-WEB.zip. Click here for file

Additional file 3 **Full-length LTR elements described in AnoTExcel**. Main structural and evolutionary characteristics of the Full-length LTR elements present in AnoTExcel. Click here for file

Additional file 4 **Nucleotide distances (p-distances) among Copia elements from *Anopheles gambiae***. Matrix with the p-distances among all the Copia elements previously described in Repbase (Copia1-5 + Mtanga) plus the Novel Copia elements described here (clu134 and 149 from AnoTExcel). Click here for file

Additional file 5 **Novel LTR elements described in AnoTExcel**. Main structural and evolutionary characteristics of four novel LTR elements in AnoTExcel. Click here for file

Additional file 6 **Full-length NLTR elements described in AnoTExcel**. Main structural and evolutionary characteristics of NLTR elements in AnoTExcel. Click here for file

Additional file 7 **Full-length Class II elements described in AnoTExcel**. Main structural and evolutionary characteristics of the Full-length Class II elements in AnoTExcel. Click here for file

Additional file 8 **MITE-like Novel elements related to unknown TEs described in AnoTExcel**. Main structural and evolutionary characteristics of MITE-like elements described in AnoTExcel. Click here for file
