# Supplementary material for: Novel transposable elements from Anopheles gambiae
Source: BMC Genomics. 2011 May 23;12:260. doi: 10.1186/1471-2164-12-260 (PMC3212995; doi:10.1186/1471-2164-12-260)
Supplement: Additional file 1 — AnoTExcel database in standalone format. It includes the links that need to be extracted to the user's computer http://exon.niaid.nih.gov/transcriptome/TE/A_gambiae/AnoTExcel-SA.zip. [file 1471-2164-12-260-S1.DOC]

**Additional file 1 - zip file - AnoTExcel database in standalone format.** It includes the links that need to be extracted to the user's computer <http://exon.niaid.nih.gov/transcriptome/TE/A_gambiae/AnoTExcel-SA.zip>
