# Supplementary material for: Novel transposable elements from Anopheles gambiae
Source: BMC Genomics. 2011 May 23;12:260. doi: 10.1186/1471-2164-12-260 (PMC3212995; doi:10.1186/1471-2164-12-260)
Supplement: Additional file 2 — Web-based links of AnoTExcel. http://exon.niaid.nih.gov/transcriptome/TE/A_gambiae/AnoTExcel-WEB.zip. [file 1471-2164-12-260-S2.DOC]

**Additional file 2 – zip file - Web-based links of AnoTExcel** <http://exon.niaid.nih.gov/transcriptome/TE/A_gambiae/AnoTExcel-WEB.zip>
